# Combined indicator assists in early recognition of retinopathy of prematurity

**DOI:** 10.1038/s41598-025-92205-3

**Published:** 2025-03-07

**Authors:** Wen Shi, Liping Zhu, Xiaori He, Shuo Wang, Cheng Wang

**Affiliations:** 1 Department of Pediatrics, The Second Xiangya Hospital, Central South University, Changsha, 410011 Hunan China; 2 Department of Ophthalmology, The Second Xiangya Hospital, Central South University, Changsha, 410011 Hunan China; 3 Department of Ophthalmology, Guilin Hospital of The Second Xiangya Hospital, Central South University, Guilin, 541001 Guangxi China; 4 Department of Neonatology, Changsha Central Hospital, University of South China, Changsha, 410028 Hunan China; 5 Department of Pediatrics, Xiangya Hospital, Central South University, Changsha, 410008 Hunan China

**Keywords:** Retinopathy of prematurity, Predictive, Combined indicator, Early recognition, Preterm infants, Diagnostic markers, Predictive markers, Risk factors

## Abstract

This research aimed to investigate the value of clinical data in preterm infants on admission for the early prediction of retinopathy of prematurity (ROP). 98 preterm infants (66 males and 32 females) with an average gestational age of 30.42 ± 1.20 weeks were included. Basic vital signs, clinical tests, and maternal information were collected at admission. Preterm infants were divided into a non-ROP group and a ROP group based on whether they eventually developed ROP. The receiver operating characteristic (ROC) curve was used to evaluate the predictive value of the above indexes and the combined indexes in the ROP of preterm infants. (1) The differences in systolic blood pressure (SBP), red blood cell count (RBC), hemoglobin (HGB), direct bilirubin (DBIL), and total bilirubin (TBIL) were statistically significant between the non-ROP group and ROP group (all *P* < 0.05). (2) RBC, HGB, DBIL, and TBIL, all of which have diagnostic value for ROP [area under curve (AUC) 0.643, 0644, 0.887, and 0.744, respectively, all *P* < 0.05]. (3) The combined indicator possessed a good diagnostic value for ROP (AUC of 0.962, *P* < 0.05), with a sensitivity and specificity of 88.64% and 91.49%, respectively. (4) Combined indicator (body temperature, body weight, heart rate, SBP, diastolic blood pressure, mean arterial pressure, RBC, HGB, red blood cell distribution width, DBIL, TBIL) has better diagnostic value for ROP than each of RBC, HGB, DBIL, and TBIL alone (Z-value 5.386, 5.475, 2.410 and 4.420, respectively, all *P* < 0.05). Combined indicator has good predictive value for ROP in preterm infants.

## Background

Retinopathy of prematurity (ROP) is a proliferative disease of the retina that occurs in premature infants and infants with low birth weight (LBW). 65.8% of infants had some degree of ROP; 81.6% for infants of less than 1000 g birth weight. Lower birth weight and gestational age had a higher incidence and severity of ROP^1^ which is the leading cause of poor vision and blindness in infants and young children. The mortality rate of LBW and premature infants has improved over the years due to improvement in medical technology, thus, the incidence of ROP has increased annually^2^. The diagnosis of ROP relies on digital fundus cameras or indirect ophthalmoscopes. However, by the time ROP is detected, the retina has already been damaged. ROP can be reduced or even prevented by early identification in infants at high risk of ROP^3^ and dynamic adjustment of their oxygen therapy or other disease management regimens. Some clinical biological markers, such as circRNAs expression profile changes in peripheral blood mononuclear cells (PBMCs)^4^, serum fructosamine^5^, complete blood count (CBC) in the first week after birth^6^, critical levels of interleukin-6 (IL-6), tumour necrosis factor-α (TNF-α) and vascular endothelial growth factor-A in cord blood^7^, serum IL-33 and endorphin levels^8^, etc., revealed some value in the early detection of ROP. The aim of this research was to investigate the relationship between vital signs, clinical examination, maternal information and ROP in preterm infants and to explore a simple and practical marker for early prediction of ROP in preterm infants.

## Methods

### Study population

49 preterm infants admitted to our Neonatal Intensive Care Unit (NICU) with a final diagnosis of ROP between Jun. 2022 and Dec. 2022 were retrospectively collected as the ROP group (34 males, 15 females, mean gestational age 30.58 ± 1.05 weeks). The non-ROP group was matched with 49 preterm infants with similar gestational age and birth weight admitted to NICU at the same time (32 males, 17 females, mean gestational age 30.25 ± 1.32 weeks). Cases in the ROP group/showed at least a demarcation line between the peripheral avascular zone and the retinal vascular endings in the posterior pole, i.e., ROP stage I. Informed consent was signed by the subjects’ legal guardians. This research was approved by the Medical Ethics Committee of The Second Xiangya Hospital, Central South University [Ethical Audi No. Study K034 (2021)] and conforms to the principles stated in the Declaration of Helsinki.

### Inclusion and exclusion criteria

Inclusion criteria: Meet the diagnostic criteria for preterm infants and ROP. Exclusion criteria: Preterm infants with complex congenital heart disease, inherited metabolic diseases, and congenital multiple malformations, such as congenital abdominal cleft, intestinal atresia, esophageal atresia, esophageal-tracheal fistula, and congenital biliary atresia; for whom it is not possible to dilate pupils to perform funduscopic examination^9,10^.

### Variables

Categorical variables: sex, body weight, body temperature, heart rate (HR), systolic blood pressure (SBP), diastolic blood pressure (DBP), mean arterial pressure (MAP), red blood cell count (RBC), hemoglobin (HGB), red blood cell distribution width (RDW), direct bilirubin (DBIL), total bilirubin (TBIL).

Continuous variables: polyembryony, weight and gestational age classification, gestational hypertension, delivery mode, gestational diabetes mellitus (GDM), and intrauterine distress.

### Fundus examination

(1) Prior to the examination, informed consent was signed by the legal guardians of the infants. Mydrin-P eye drops (compound tropicamide eye drops containing 50 mg tropicamide and 50 mg phenylephrine hydrochloride; Santen Pharmaceutical Co., Ltd, Osaka, Japan) were then applied 2–3 times in both eyes at 15-minute intervals. After the pupil dilated, a digital fundus camera (SW-8000P, Tianjin Suowei Electronic Technology, China) was used to perform fundus examination, taking retinal imaging in 5 directions including midline, up, down, left, and right. The fundus photos were sent to experienced ophthalmologists.

(2) Results record: Ophthalmologists described the development of retinal blood vessels based on fundus photos and recorded the zoning, staging, and presence of bleeding, neovascularization, retinal detachment, or not. ROP diagnosis and classification was conducted according to the ROP screening guideline published by the Chinese Ophthalmological Society and the International Classification of Retinopathy of Prematurity, Third Edition^9–11^.

### Statistical analysis

If the continuous variable is normally distributed, it is expressed as mean ± SD; otherwise, it is expressed as the quartile M (P25, P75). Categorical variables were expressed in frequency or as a percentage. Chi-square test (categorical variables), Student’s t-test (normal distribution), or Mann-Whitney U-test (skewed distribution) were used to analyze differences between non-ROP group and ROP group. A receiver operating characteristic (ROC) curve was used to evaluate the diagnostic efficacy of different indicators for ROP. Logistic regression was used to combine the diagnostic efficacy of multiple indicators. The area under the curve (AUC) was compared using the Z-test. AUC = 1 denotes perfect prediction. 0.85 < AUC ≤ 0.95 represents excellent prediction. 0.7 < AUC ≤ 0.85 indicates moderate prediction. 0.5 < AUC ≤ 0.7 represents low prediction. *P*-values < 0.05 (two-sided) were considered statistically significant.

## Results

### Study population comparison between the non-ROP group and ROP group

There were 98 preterm infants included in this study, 66 males and 32 females, with a mean gestational age of 30.42 ± 1.20 weeks. SBP, RBC, HGB, DBIL, and TBIL in the ROP group were higher than those in the non-ROP group (*P* < 0.05) (Table 1).

**Table 1:** Study population comparison between non-ROP group and ROP group [Mean ± SD, n (%)]

	non-ROP group	ROP group	Standardize diff.	*P*-value
N	49	49		
Sex			0.09 (-0.31, 0.48)	0.667
Male	32 (65.31)	34 (69.39)		
Female	17 (34.69)	15 (30.61)		
Polyembryony			0.30 (-0.10, 0.70)	0.145
No	34 (69.39)	27 (55.10)		
Yes	15 (30.61)	22 (44.90)		
Weight and gestational age classification			0.15 (-0.25, 0.54)	0.770
Appropriate for gestation age (AGA)	43 (87.76)	45 (91.84)		
Large for gestational age (LGA)	2 (4.08)	1 (2.04)		
Small for gestational age (SGA)	4 (8.16)	3 (6.12)		
Mode of delivery			0.16 (-0.23, 0.56)	0.424
Vaginal delivery	10 (20.41)	7 (14.29)		
Cesarean Section	39 (79.59)	42 (85.71)		
Intrauterine distress			0.30 (-0.10, 0.69)	0.146
No	41 (83.67)	35 (71.43)		
Yes	8 (16.33)	14 (28.57)		
Hypertension in pregnancy (HIP)			0.38 (-0.02, 0.78)	0.062
No	44 (89.80)	37 (75.51)		
Yes	5 (10.20)	12 (24.49)		
Gestational diabetes			0.18 (-0.22, 0.57)	0.381
No	36 (73.47)	32 (65.31)		
Yes	13 (26.53)	17 (34.69)		
Body temperature (℃)	36.16 ± 0.56	36.29 ± 0.56	0.23 (-0.17, 0.63)	0.258
Body weight (g)	1433.78 ± 296.77	1400.92 ± 292.37	0.11 (-0.28, 0.51)	0.582
HR (bpm)	145.92 ± 13.43	141.76 ± 14.95	0.29 (-0.11, 0.69)	0.150
SBP (mmHg)	49.92 ± 7.22	53.84 ± 9.90	0.45 (0.05, 0.86)	0.029
DBP (mmHg)	29.21 ± 5.89	29.39 ± 6.62	0.03 (-0.37, 0.43)	0.888
MAP (mmHg)	37.64 ± 6.77	39.52 ± 8.60	0.24 (-0.17, 0.66)	0.247
RBC (×10^12^)	4.08 ± 0.55	4.31 ± 0.61	0.40 (0.00, 0.80)	0.049
HGB (g/L)	152.98 ± 22.39	163.55 ± 25.90	0.44 (0.04, 0.84)	0.033
DBIL (µmol/L )	8.40 ± 1.90	14.00 ± 4.32	1.68 (1.22, 2.14)	< 0.001
TBIL (µmol/L)	90.52 ± 26.12	117.59 ± 28.28	0.99 (0.57, 1.41)	< 0.001
RDW (fL)	16.89 ± 3.53	16.54 ± 1.31	0.13 (-0.27, 0.53)	0.520

### ROC for single indicator on ROP

**Fig. 1:**
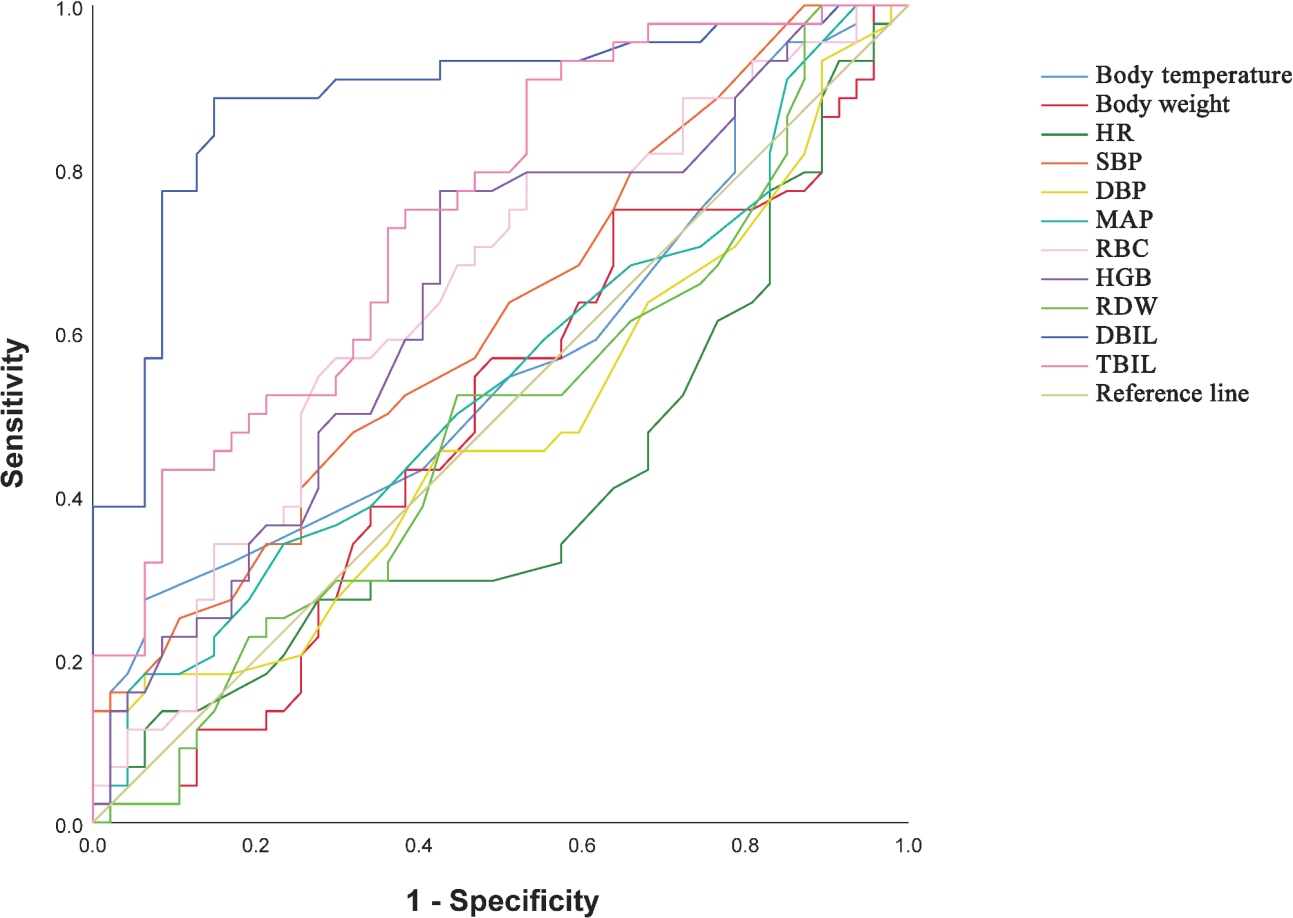
ROC for single indicator on ROP.

RBC and HGB had a low predictive efficacy for ROP (AUC of 0.643 and 0.644, respectively, all *P* < 0.05); DBIL had an excellent predictive efficacy for ROP (AUC of 0.887, with sensitivity and specificity of 88.64% and 85.11%, respectively, *P* < 0.001). TBIL had a moderate predictive efficacy for ROP (AUC 0.744, with sensitivity and specificity of 90.91% and 46.81%, respectively, *P* < 0.001) (Table 2; Fig. 1).

**Table 2:** ROC for single indicator on whether or not ROP.

Indicator	AUC	95%CI	*P*-value	Cut-off value	Sensitivity (%)	Specificity (%)	Positive likelihood ratio	Negative likelihood ratio	Jordon index
body temperature (℃)	0.562	0.442–0.682	0.307	36.65	27.27	93.62	4.27	0.78	0.21
Body weight (g)	0.491	0.371–0.611	0.880	1265.00	75.00	36.17	1.18	0.69	0.11
HR (bpm)	0.406	0.286–0.525	0.061	158.50	13.64	91.49	1.60	0.94	0.05
SBP (mmHg)	0.616	0.501–0.731	0.057	53.50	47.73	68.09	1.50	0.77	0.16
DBP (mmHg)	0.477	0.357–0.598	0.709	36.50	18.18	93.62	2.85	0.87	0.12
MAP (mmHg)	0.541	0.422–0.661	0.497	47.50	18.18	93.62	2.85	0.87	0.12
RBC (×10^12^)	0.643	0.529–0.757	0.019	4.31	56.82	70.21	1.91	0.62	0.27
HGB (g/L)	0.644	0.530–0.758	0.018	151.50	77.27	57.45	1.82	0.40	0.35
RDW (fL)	0.488	0.368–0.608	0.849	13.50	100.00	10.64	1.12	0.00	0.11
DBIL (µmol/L)	0.887	0.815–0.959	0.000	9.55	88.64	85.11	5.95	0.13	0.74
TBIL (µmol/L)	0.744	0.645–0.844	0.000	87.00	90.91	46.81	1.71	0.19	0.38

**Table 3:** Comparison of different ROCs.

	Area difference under the curve	95%CI	Z-value	*P*-value
Combined indicator vs. RBC	0.319	0.203–0.435	5.386	0.001
Combined indicator vs. HGB	0.318	0.204–0.432	5.475	0.001
Combined indicator vs. DBIL	0.075	0.014–0.136	2.410	0.016
Combined indicator vs. TBIL	0.218	0.121-0.315	4.420	0.001

### ROC for combined indicator on whether or not ROP

**Fig. 2:**
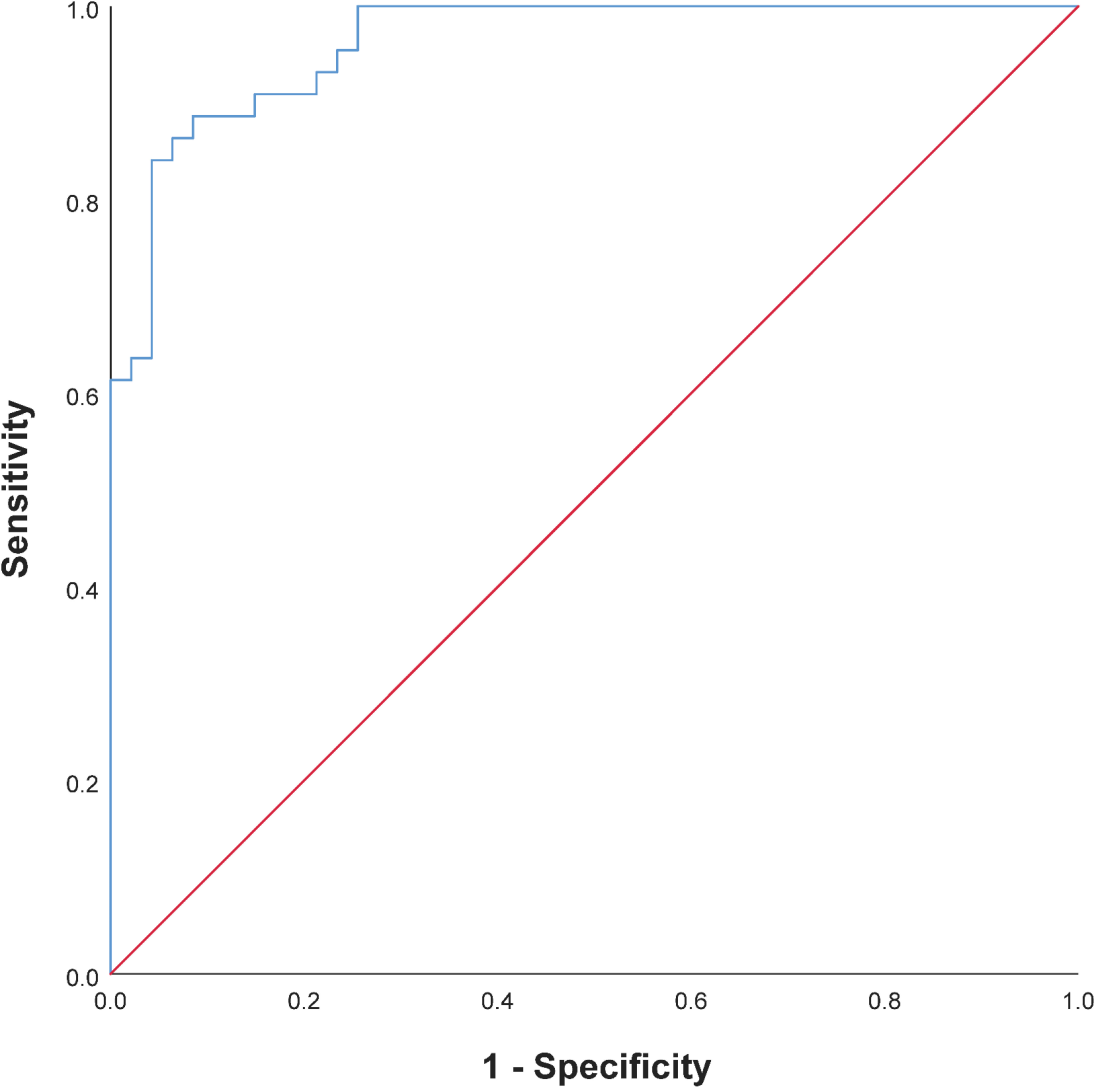
ROC for Combined indicator on ROP.

To explore whether the predictive value of ROP can be further improved, we combined the 11 indicators listed in Table 2 (specific indicators are listed, such as body temperature, body weight, HR, SBP, DBP, MAP, RBC, HGB, RDW, DBIL, TBIL). The combined indicator has an excellent predictive value for ROP (AUC = 0.962, 95%CI = 0.930–0.995, *P*-value = 0.017, cut-off value = 0.57, sensitivity = 88.64%, specificity = 91.49, positive likelihood ratio = 91.49, negative likelihood ratio = 0.12, jordon index = 0.80) (Fig. 2).

### Comparison of different ROCs

**Fig. 3:**
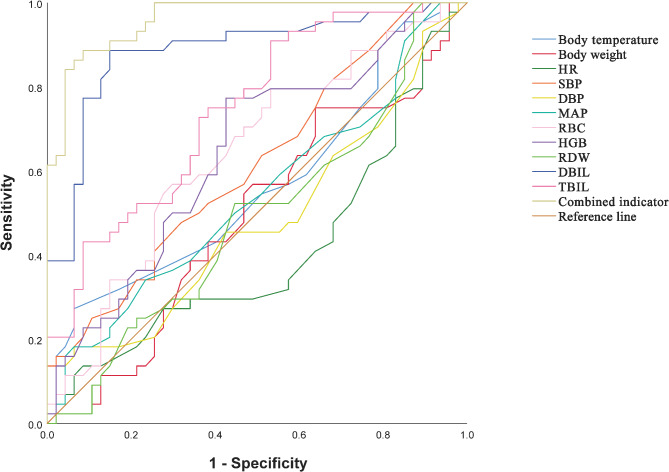
ROC for all indicators on ROP.

To compare whether the 11 combined indicators (body temperature, body weight, HR, SBP, DBP, MAP, RBC, HGB, RDW, DBIL, TBIL) were superior to the predictive value of RBC, HGB, TBIL, and DBIL for ROP, a comparison of multiple ROCs was performed. The AUC of the combined index was significantly greater than that of RBC, HGB, TBIL, and DBIL, thereby providing better predictive value. (Z = 5.386, 5.475, 2.410, 4.420, all *P*<0.05). Thus, the predictive value of the combined indicator for ROP is better than the remaining four indicators (Table 3; Fig. 3).

## Discussion

Early detection and diagnosis of ROP can prevent retinal detachment and blindness. Clinical screening of newborn infants for ocular disease has been undertaken with a focus on ROP. Some biomarkers have been reported in the literature which revealed some value in predicting retinopathy of prematurity, while they may be difficult to obtain or have limited predictive value. A few researchers have used possible risk factors such as gestational age, birth weight, sepsis, respiratory distress syndrome, abdominal bleeding, continuous positive pressure ventilation, oxygen saturation, apnoea, number and duration of blood transfusions, oxygen therapy and phototherapy to predict the occurrence of ROP^12^, but the cut-off time of the indicators were long or the predictive accuracy was low, so they were still not good early predictions of ROP. Therefore, there is still a demand to explore the clinical utility of biomarkers as more effective predictive tools. The indicators used in our study were common indicators (including body temperature, weight, HR, SBP, DBP, MAP, RBC, HGB, RDW, DBIL, and TBIL), all of which were available at the time of admission, which allowed us to anticipate the occurrence of ROP in neonates in the first place, and thus pay attention to early intervention.

The results of this research showed that RBC, HGB, DBIL and TBIL had predictive value for ROP. RBC and HGB are normally involved in oxygen transport in the body. Anaemia caused by reduced RBC and HGB leads to reduced oxygen levels in the blood, and retinal blood vessels are very sensitive to oxygen supply. They require sufficient oxygen to maintain normal vascular development and function. If the oxygen supply is inadequate, the vascular endothelial cells can be damaged, affecting the normal growth and branching of blood vessels^13^. Anaemia can also cause an imbalance of angiogenic factors, abnormal blood flow and hypoperfusion, oxidative stress and inflammatory responses^14^. Anaemia indicates that oxygen delivery to the retina may be compromised, thereby increasing the risk of ROP. However, those two indicators are only indicative of possible anaemia, which may explain the low predictive value.

The predictive value of DBIL and TBIL for ROP may be due to the fact that they are measures of bilirubin levels, which are mainly produced by the liver and excreted via the biliary tract. Therefore, they partially reflect the function of the hepatobiliary system^15–17^. The immature development of the hepatobiliary system in premature infants may lead to bilirubin metabolism disorders, and high levels of DBIL and TBIL may indicate abnormal function of the hepatobiliary system. Bilirubin is an endogenous antioxidant that reacts with oxygen free radicals, thereby interfering with their activity^18,19^. However, the antioxidant effects of bilirubin are not absolute and its effectiveness depends on many factors. Elevated bilirubin can sometimes be associated with increased production of oxygen free radicals. For example, the body may produce more free radicals in certain disease states or under stressful conditions, and elevated bilirubin may not compensate for the increase in free radicals. In this case, high bilirubin levels also indicate a state of high oxygen free radicals. Excessive oxygen free radicals may cause early damage to retinal vascular endothelial cells, affect vascular development and function, and increase the risk of ROP^20^. Hoppe et al.^21^ reported that reducing hypoxia-inducing factor (HIF) may mitigate the damaging effects on the retina.

The main finding of this research is that combining indicators that do not appear to have predictive value improves the predictive value of ROP. Abnormal body temperature usually indicates infection or other health problems. Infection and inflammation can damage the vascular endothelial cells of the retina and affect the normal development of blood vessels, which is associated with the development of ROP. Lyu et al.^22^ reported that neonatal hypothermia was associated with severe retinopathy in preterm infants. In addition, there was a U-shaped relationship between admission temperature and adverse neonatal outcomes. The lowest incidence of adverse outcomes occurred when the admission temperature was between 36.5 °C and 37.2 °C. In addition, LBW is one of the characteristics of preterm infants, which can lead to immature retinal development and fragile blood vessels, making preterm infants more prone to retinal vascular abnormalities, which is a major risk factor for ROP. Ozdemir et al.^23^ reported that birth weight and gestational age were positively correlated with lens thickness, vitreous length and axial length (*r* > 0.5).

Changes in HR and BP reflect the functional status of the cardiovascular system. A dysfunctional cardiovascular system may affect blood flow to the retina, with hypotension causing retinal ischaemia and increasing the risk of vascular injury, and hypertension inhibiting retinal vessel growth. Kistner et al.^24^ reported that preterm infants with severe abnormalities in retinal vascular development during the neonatal period may have an increased risk of elevated blood pressure in adulthood. RDW reflects the degree of variation in RBC size. A large RDW may indicate abnormal RBC morphology and function, which may affect oxygen delivery and blood supply to the retina.

To improve the ability to predict ROP, the combined indicator takes into account several potential risk factors for ROP and integrates them using a regression equation. This allows analysis of general data at the time of admission. Park et al.^25^ reported that elevated levels of IL-6 and C5a in cord plasma can be used as independent indicators to predict severe ROP and laser therapy, respectively. The combined model can predict the progression of ROP with good accuracy. The combined indicator in this research is convenient and practical for clinical collection. They can also be used as predictive biological markers for routine application in ROP.

## Strengths and limitations

This study yielded good ROP prediction ability by fitting general data on preterm hospital admission. The indexes involved are straightforward, practical, and cost-effective. Moreover, the relatively small sample size and single-center data sources are the limitations of our study, which need to be validated in a larger cohort and in multi-center facilities. Moreover, the generality of our findings may be influenced by changes in ethnic and demographic distributions. Prospective studies should address these limitations to establish the availability of broader joint indicators in different clinical settings.

## Conclusions

The combined indicator has an excellent predictive value for ROP, and the universality and cost-effectiveness of the indicators involved allow pediatricians at the primary level to better diagnose and manage ROP at an early stage.

## Data Availability

The datasets used and/or analyzed during the current study are available from the corresponding author upon reasonable request.
